# Prediction of length and diameter of hamstring tendon autografts for knee ligament surgery in Caucasians

**DOI:** 10.1007/s00167-015-3678-5

**Published:** 2015-06-28

**Authors:** Rob P. A. Janssen, Maria J. F. van der Velden, Marijn van den Besselaar, Max Reijman

**Affiliations:** Orthopaedic Center Máxima, Máxima Medical Center, Postbus 90052, 5600 PD Eindhoven, The Netherlands

**Keywords:** Hamstring, Tendon, Autograft, Length, Diameter, Knee ligament reconstruction, ACL, Multiligament

## Abstract

**Purpose:**

Prediction of hamstring tendon autograft size facilitates preoperative planning of knee ligament surgery and may reduce the need for allografts in complex knee reconstructions. The aim of this study was to analyse whether length and diameter of hamstring tendon autografts can be predicted preoperatively with anthropometric parameters and patient characteristics.

**Methods:**

In this observational study, 725 consecutive Caucasian patients scheduled for ACL reconstruction were included. Preoperatively gender, age, height and weight were recorded. After harvest, tendon lengths of both gracilis and semitendinosus tendons were measured. Diameter of the final four-strand hamstring autograft was recorded. Relationship between length and diameter of tendon grafts and different anthropometric parameters were assed by linear and logistic regression analyses.

**Results:**

Mean lengths of the semitendinosus and gracilis tendon autografts were 28.9 ± 3.1 and 27.7 ± 3.0 cm, respectively. Length of the gracilis and semitendinosus grafts was independently related to patient height. Female gender was correlated with smaller graft diameter. One in nine female patients had a diameter <8 mm. The ratio in men was 1 in 36.

**Conclusion:**

Hamstring autograft length and size can be predicted in Caucasians. Length of the gracilis and semitendinosus tendons was related to patient height. Smaller graft diameter was related to female gender.

**Level of evidence:**

II.

## Introduction

Hamstring autografts have become increasingly popular for knee ligament reconstructions [22]. In 2012, 95 % of the primary anterior cruciate ligament (ACL) reconstructions in Sweden were performed with hamstring tendon autografts [14]. In a recent review, Samuelsson et al. [30] stated that hamstring tendons, compared to bone-patella tendon-bone autografts, put the surgical skills of the orthopedic surgeon to test because it is not possible to preoperatively assess the graft dimensions of the tendon. Complex knee ligament reconstructions require specific tendon length and diameter depending on the fixation method, graft preparation technique and type of reconstruction [6, 7, 11, 15, 20, 21, 25].

The question arises whether hamstring autograft tendons will be of sufficient length and diameter for knee ligament surgery. Various authors have analysed the predictability of hamstring tendon dimensions by anthropometric parameters [2, 4, 5, 10, 16, 18, 24–27, 29, 31, 32, 35–38]. Some studies analysed graft diameter [2, 4, 18, 25, 27, 35, 37]. Studies that focused on hamstring tendon length used small study populations or human cadavers [5, 10, 16, 24, 26, 29, 31, 32, 36]. Only Xie et al. [38] studied a larger population of 235 Chinese Han patients. Hamstring tendon length appears to be longer in Caucasian patients in comparison with Chinese patients [5]. Length of hamstring tendon autografts has not been studied in a large Caucasian population.

Prediction of length and diameter of hamstring tendon autografts is clinically useful. It may reduce the need for expensive allografts in complex knee ligament surgery [8, 23]. Autografts enhance the quality of reconstructions with regard to graft rupture and knee stability compared to allografts [1, 3, 9, 13, 19, 28, 33]. Furthermore, prediction of hamstring autograft tendon dimension is beneficiary for complex knee ligament reconstructions in countries where allografts are not available.

The primary aim of the present study is to analyse the preoperative predictability of hamstring tendon length and diameter with anthropometric parameters and patient characteristics in a large Caucasian population. The hypothesis is that length and diameter of hamstring tendon autografts can be predicted by anthropometric parameters.

## Materials and methods

In this observational study, 725 consecutive Caucasian patients with ACL rupture, scheduled for ACL reconstruction between October 2007 and January 2014, were eligible for inclusion. All patients undergoing primary or revision ACL reconstruction with four-strand hamstring tendon autografts were included. Exclusion criteria were ACL reconstruction with other auto- or allografts, previous ipsilateral hamstring tendon harvest, previous limb amputation or congenital limb deficiency that would affect total body weight, neuromuscular disorders and non-Caucasian race.

Preoperatively gender, age, height and weight were recorded. Two orthopedic surgeons (RJ and MB) performed all procedures using the same hamstring tendon harvesting technique. Both gracilis and semitendinosus tendons were harvested for ACL reconstruction. The hamstring tendon harvesting technique has been described in detail in a previous publication [12].

Both tendons were prepared in a standardized fashion as a four-strand ACL graft. Muscle tissue was removed from the tendon. The available length of each tendon was measured with a ruler and recorded in cm, rounded off to the nearest 0.5 cm. Each tendon end was sutured with a no. 1 absorbable suture. The diameter of the four-strand hamstring graft was measured by sizing tubes (Biomet, Warsaw, IN, USA) calibrated to 1 mm, range 7–9 mm.

The correlation was evaluated between length and diameter of harvested hamstring tendons and gender, age, height and weight of the patient.

The study was approved by an independent medical ethical committee (METC 2014-30; Máxima Medical Center, Eindhoven, The Netherlands).

### Statistical analysis

Linear regression analyses were used to analyse the predictability of hamstring tendon autograft length. For the diameter of hamstring tendon autografts (cut-off point of <8 mm), logistic regression analyses were performed.

Length of the hamstring tendon autografts and diameter of the four-strand ACL grafts were used as dependent variables, and the anthropometric parameters and patient characteristics as independent variables. Univariable and multivariate analyses were performed by the enter method. The following anthropometric parameters and patient characteristics were analysed: gender, age, height and weight.

Most current knee ligament reconstructions require graft lengths of 20–28 cm [11, 15, 17]. Therefore, tendon length cut-off points of 21 and 28 cm were determined.

Predictability of hamstring tendon autograft length of <21 and <28 cm was analysed by assessing the above-mentioned anthropometric parameters and patient characteristics. Significance was set at ≤0.05 in all analyses. IBM SPSS Statistics version 19.0 (IBM, Armonk, New York) was used for statistical analysis.

## Results

Baseline characteristics of the study population are shown in Table 1. Eighteen patients had missing data (respectively, height, weight, gracilis length, semitendinosus length and graft diameter in 8, 11, 4, 2 and 2 patients). Some patients had more than one missing value.

**Table 1:** Patient characteristics

	Total (*n* = 725)
Age (year)	28.7 (±10.6)
Gender [% female (*n*)]	35.4 (257)
Weight (kg)	76.5 (±13.4)
Height (cm)	177.3 (±9)
BMI (kg/m^2^)	24.3 (±3.5)

Data are presented as mean (standard deviation), unless otherwise indicated

*n* number, *BMI* body mass index

Mean lengths of the semitendinosus and gracilis tendon autograft were 28.9 ± 3.1 standard deviation (SD) cm and 27.7 ± 3.0 cm SD, respectively. Two patients (0.3 %) had a semitendinosus tendon length shorter than 21 cm. Twelve patients (1.7 %) had gracilis tendons shorter than 21 cm. A total of 42 patients (5.8 %) had graft diameters ≤7 mm, 359 patients (49.7 %) had graft diameters of 8 mm, and 322 patients (44.5 %) diameters ≥9 mm.

Length of both the gracilis and semitendinosus tendon was correlated to patient height (Table 2). A regression coefficient of 0.16 signifies that an increase of 1 cm in patient height is correlated with an increase of 0.16 cm in gracilis length. Because of the limited number of patients with tendon autografts <21 cm, assessment of the relationship between this cut-off point and anthropometric parameters and patient characteristics was not performed. With regard to tendon lengths <28 cm, shorter patients more frequently had gracilis and semitendinosus tendon autografts <28 cm (Table 3).

**Table 2:** Predictors of tendon length

	Univariable		Multivariate	
	Regr. co. (95 % CI)	*P* value	Regr. co. (95 % CI)	*P* value
Gracilis				
Age	−0.01 (−0.03; 0.01)	n.s.	–	–
Gender (female)	−1.9 (−2.3; −1.4)	<0.001	0.06 (−0.49; 0.62)	n.s.
Weight	0.06 (0.04; 0.07)	<0.001	−0.005 (−0.02; 0.01)	n.s.
Height	0.15 (0.13; 0.17)	<0.001	0.16 (0.13; 0.19)	<0.001
Semitendinosus				
Age	−0.01 (−0.03; 0.01)	n.s.	–	–
Gender (female)	−2.4 (−2.8; −1.9)	<0.001	−0.03 (−0.55; 0.49)	n.s.
Weight	0.07 (0.06; 0.09)	<0.001	−0.01 (−0.02; 0.01)	n.s.
Height	0.19 (0.17; 0.21)	<0.001	0.20 (0.17; 0.23)	<0.001

*Regr. co.* regression coefficients, *95* *% CI* 95 % confidence interval, *n.s.* non-significant

**Table 3:** Categories of body height related to tendon length

Height (cm)	Gracilis		Semitendinosus	
	<28 cm [% (*n*)]	≥28 cm [% (*n*)]	<28 cm [% (*n*)]	≥28 cm [% (*n*)]
≤160 (*n* = 33)	87.9 (29)	12.1 (4)	81.8 (27)	18.2 (6)
161–165 (*n* = 47)	78.7 (37)	21.3 (10)	61.7 (29)	38.3 (18)
166–170 (*n* = 100)	68.0 (68)	32.0 (32)	55.0 (55)	45.0 (45)
171–180 (*n* = 280)	46.4 (130)	53.6 (150)	30.7 (86)	69.3 (194)
181–190 (*n* = 213)	23.7 (50)^a^	76.3 (161)^a^	13.6 (29)	86.4 (184)
≥191 (*n* = 42)	16.7 (7)	83.3 (35)	7.1 (3)	92.9 (39)
Total (*n* = 715)	45.0 (321)^a^	55.0 (392)^a^	32.0 (229)	68.0 (486)

*n* number

^a^Two missing values gracilis length

A correlation was found between gender and graft diameter <8 mm (Table 4). Table 5 shows that women more often had a graft diameter <8 mm in comparison with men.

**Table 4:** Predictors of diameter graft <8 mm

	Univariable		Multivariate	
	OR (95 % CI)	*P* value	OR (95 % CI)	*P* value
Age	0.98 (0.95; 1.01)	n.s.	–	–
Gender (female)	4.5 (2.3; 8.7)	<0.001	4.5 (1.9; 11.0)	0.001
Weight	0.95 (0.93; 0.98)	0.001	0.97 (0.93; 1.01)	n.s.
Height	0.96 (0.93; 0.99)	0.019	1.04 (0.99; 1.10)	n.s.

*OR* odds ratios, *95* *% CI* 95 % confidence interval, *n.s.* non-significant

**Table 5:** Gender related to diameter graft

	<8 mm [% (*n*)]	8 mm [% (*n*)]	>8 mm [% (*n*)]
Male (*n* = 467)	2.8 (13)	36.8 (172)	60.4 (282)
Female (*n* = 256)	11.3 (29)	73.1 (187)	15.6 (40)
Total (*n* = 723)	5.8 (42)	49.7 (359)	44.5 (322)

Two missing values graft diameter

*n* number

## Discussion

The most important finding of the present study is that length and diameter of hamstring tendon autografts can be predicted by anthropometric parameters in patients of Caucasian race. Length of gracilis and semitendinosus tendons was independently related to patient height. Smaller graft diameter was independently related to female gender.

### Hamstring autograft length prediction

In the present study, hamstring tendon length of both semitendinosus and gracilis autograft tendons was correlated to patient height. A similar correlation was found by other studies with smaller and/or non-Caucasian populations [5, 10, 24, 29, 32, 36, 38]. Chiang et al. [5] studied a group of 100 Chinese patients and found a significant correlation between height and length of both semitendinosus and gracilis tendons after multiple linear regression analysis. The authors compared their data to the study population by Treme et al. and concluded that Caucasian patients had significantly longer hamstring tendons compared to the Chinese Han population [5, 36]. This conclusion on racial difference by Chiang et al. [5] may be questionable since Treme et al. [36] did not specify the race of their 50 consecutive patients. Xie et al. concluded that height and weight were the best predictors for hamstring tendon length in men, whereas only height was a predictor in women. Analysis was by simple linear regression [38]. Although the present study showed weight to be a predictor for semitendinosus and gracilis tendon length in a univariable logistic regression analysis, this relationship was explained by patient height as demonstrated in the multivariate logistic regression analysis.

Gender has been associated with hamstring graft length [36, 38]. Xie et al. [38] described that women had significantly shorter hamstring tendons than men. Treme et al. [36] found that women had significantly smaller and shorter grafts compared to men. Other authors have confirmed these findings [5, 24]. The present study found gender to be a predictor of semitendinosus and gracilis tendon length in the univariable logistic regression analysis. However, this relationship can also be explained by patient height. Strength of the present study is the large sample size and level of statistical analysis. This may explain the variance in significance found in the literature for anthropometric predictors of graft length.

Current anatomic ACL reconstructions and fixation techniques allow the use of multiple-stranded hamstring autografts with a minimal tendon length of 21 cm [17]. Pichler et al. [26] studied the length of harvested hamstring tendons. The shortest harvested semitendinosus tendon was at least 20 cm long, and 11 % of gracilis tendons were shorter than 20 cm. Leg length was the only anthropometric parameter in their cadaveric study. In contrast, the present study demonstrated that only two patients (0.3 %) had semitendinosus tendon length <21 cm. The gracilis tendon length was <21 cm in 12 patients (1.7 %). Hamstring tendon length was predictable, and almost always of sufficient length (≥21 cm) for ACL and MPFL reconstructions in a Caucasian population [11, 17].

Longer grafts may be necessary for complex knee ligament surgery [15, 17]. Papastergiou et al. [24] analysed the predictability of semitendinosus tendon length by anthropometric parameters. Seventy-nine percent of harvested semitendinosus tendons were ≥28 cm. However, length of semitendinosus tendons was <28 cm in 43.8 % of female patients. Patients shorter than 167 cm were at highest risk for semitendinosus tendons <28 cm [24]. This is comparable to the results of the present study; height ≥170 cm showed a greater probability of semitendinosus and/or gracilis tendons ≥28 cm. The comparable patient height in both studies (176.3 ± 8 cm [24] and 177.3 ± 9 cm, respectively) could explain these similar results. The present study showed that hamstring tendon length was predictable for complex knee ligament surgery in Caucasians.

### Hamstring autograft diameter prediction

The present study has shown that the diameter of a four-strand hamstring tendon autograft is significantly correlated to gender. One in nine female patients had a diameter <8 mm. The ratio in men was 1 in 36. The correlation between gender and graft diameter has been described in previous studies [2, 18, 25–27, 35–38].

Park et al. [25] concluded that graft diameter <8 mm led to significantly more failures. In younger patients, higher failure rates of ACL reconstructions with graft diameter ≤8 mm have been described [20, 21]. In the present study, graft diameter was <8 mm in 2.8 % of men and 11.3 % of women. In contrast, Ma et al. [18] found graft diameter <8 mm in 18.4 % of men and 42.3 % of women. Pinheiro et al. [27] also described a larger percentage of graft diameters <8 mm compared to the present study (18.5 % of men and 66.7 % of women). These authors also used a four-strand hamstring tendon autograft. However, it should be noted that mean patient height in the studies by Ma et al. [18] and Pinheiro et al. [27] was shorter in comparison with the present study (167.3 ± 4, 170.0 ± 10 and 177.3 ± 9 cm, respectively). This difference in height could explain the differences in graft diameter between the studies.

Ma et al. [18] also correlated height to graft diameter in a multivariate regression analysis. Men had significantly larger grafts than women (8.1 ± 0.8 vs. 7.5 ± 0.6 mm, respectively). Height was a specific predictor solely in men. In women, none of the preoperative measures were predictors for graft diameter [18]. Similarly, Papastergiou et al. [24] did not find any significant predictor for graft diameter in women. In their retrospective study of 61 consecutive patients (46 men, 16 women), the definition of adequate size graft was ≥7 mm. The majority of patients (90 %) had adequate grafts, and only 10 % of patients had grafts <7 mm. Stratified for gender, 25 % (all women) had graft sizes <7 mm. Women were significantly shorter and lighter than men. Their hamstring grafts were also shorter with smaller diameters compared to men [24]. In the present study, adequate graft diameter was defined as ≥8 mm. Therefore, the results of the present study cannot be compared to the data by Papastergiou et al. [24]. Other authors have confirmed the correlation between height and graft diameter [4, 18, 24, 25, 27, 32, 35, 37, 38].

Several authors have described correlations between graft diameter and body weight [31, 35, 37, 38] and leg length [31]. In these studies, however, statistical limitations, small sample sizes and women-to-men odds ratios may explain the different predictors for graft diameter compared to the present study. In the latter, univariable linear regression analysis showed that gender, height and weight were significantly correlated to graft diameter. However, only gender was correlated to graft diameter after multivariate linear regression analysis in this large Caucasian population.

There are several limitations to the present study. Leg length was not measured and could not be used as parameter to predict autograft tendon length. Another limitation was the missing data in 18 patients. Furthermore, height and weight were not specifically measured but self-reported by patients. This could limit the accuracy of the measurements. Nevertheless, Spencer et al. [34] assessed the validity of self-reported height and weight by comparison with measured height and weight and concluded that self-reported data are valid for identifying relationships in epidemiological studies. The harvest technique in the study could have left a remnant tendon part after harvest. Pichler et al. [26] researched this possible phenomenon in a cadaver study and concluded that insufficient tendon length was mainly caused by anatomic variations rather than tendon harvesting technique. Another limitation of the present study is the fact that ACL reconstruction only allowed sizing by 1-mm intervals instead of 0.5-mm intervals. This might have led to an overestimation of the amount of larger diameter grafts.

The clinical relevance of the present study is that prediction of hamstring tendon length and graft diameter allows better preoperative planning for complex knee ligament surgery and may reduce the necessity of allografts. This reduces surgical costs [8, 23] and increases the quality of ligament reconstructions with regard to possible graft rupture and postoperative stability [1, 3, 9, 13, 19, 28, 33]. Furthermore, knee ligament reconstructions may be performed with greater confidence in countries where allografts are not available.

## Conclusion

Length and diameter of hamstring autograft tendons can be predicted by anthropometric parameters in Caucasians. Length of gracilis and semitendinosus tendons is related to patient height. Smaller graft diameter is related to female gender.

## References

[1] Barrett GR, Luber K, Replogle WH, Manley JL (2010). Allograft anterior cruciate ligament reconstruction in the young, active patient: Tegner activity level and failure rate. Arthroscopy.

[2] Boisvert CB, Aubin ME, DeAngelis N (2011). Relationship between anthropometric measurements and hamstring autograft diameter in anterior cruciate ligament reconstruction. Am J Orthop.

[3] Borchers JR, Pedroza A, Kaeding C (2009). Activity level and graft type as risk factors for anterior cruciate ligament graft failure: a case–control study. Am J Sports Med.

[4] Celiktas M, Golpinar A, Kose O, Sutoluk Z, Celebi K, Sarpel Y (2013). Prediction of the quadruple hamstring autograft thickness in ACL reconstruction using anthropometric measures. Acta Orthop Traumatol Turc.

[5] Chiang ER, Ma HL, Wang ST, Hung SC, Liu CL, Chen TH (2012). Hamstring graft sizes differ between Chinese and Caucasians. Knee Surg Sports Traumatol Arthrosc.

[6] Claes S, Vereecke E, Maes M, Victor J, Verdonk P, Bellemans J (2013). Anatomy of the anterolateral ligament of the knee. J Anat.

[7] Conte EJ, Hyatt AE, Gatt CJ, Dhawan A (2014). Hamstring autograft size can be predicted and is a potential risk factor for anterior cruciate ligament reconstruction failure. Arthroscopy.

[8] Cooper MT, Kaeding C (2010). Comparison of the hospital cost of autograft versus allograft soft-tissue anterior cruciate ligament reconstructions. Arthroscopy.

[9] Engelman GH, Carry PM, Hitt KG, Polousky JD, Vidal AF (2014). Comparison of allograft versus autograft anterior cruciate ligament reconstruction graft survival in an active adolescent cohort. Am J Sports Med.

[10] Filho ES, Sampaio EB, Namba M, Silva JL, Albano M, Rocha LE, Agulham MA, Cunha LA (2010). Is it possible to predict the length of knee flexor tendons by anthropometry?. Rev Col Bras Cir.

[11] Golant A, Quach T, Rosen JE (2014). Medial patellofemoral ligament reconstruction with a looped semitendinosus tendon, using knotless anchor fixation on the patella and hybrid fixation on the femur. Arthrosc Tech.

[12] Janssen RP, van der Velden MJ, Pasmans HL, Sala HA (2013). Regeneration of hamstring tendons after anterior cruciate ligament reconstruction. Knee Surg Sports Traumatol Arthrosc.

[13] Kaeding CC, Aros B, Pedroza A, Pifel E, Amendola A, Andrish JT, Dunn WR, Marx RG, McCarty EC, Parker RD, Wright RW, Spindler KP (2011). Allograft versus autograft anterior cruciate ligament reconstruction: predictors of failure from a MOON prospective longitudinal cohort. Sports Health.

[14] Kvist J, Kartus J, Karlsson J, Forssblad M (2014). Results from the Swedish national anterior cruciate ligament register. Arthroscopy.

[15] Laprade RF, Wijdicks CA (2012). Surgical technique: development of an anatomic medial knee reconstruction. Clin Orthop Relat Res.

[16] Limitlaohaphan C, Kijkunasatian C, Saitongdee P (2009). Length of semitendinosus and gracilis tendons and the relationship of graft length and leg length. J Med Assoc Thai.

[17] Lubowitz JH (2012). All-inside anterior cruciate ligament graft link: graft preparation technique. Arthrosc Tech.

[18] Ma CB, Keifa E, Dunn W, Fu FH, Harner CD (2010). Can pre-operative measures predict quadruple hamstring graft diameter?. Knee.

[19] Macaulay AA, Perfetti DC, Levine WN (2012). Anterior cruciate ligament graft choices. Sports Health.

[20] Magnussen RA, Lawrence JT, West RL, Toth AP, Taylor DC, Garrett WE (2012). Graft size and patient age are predictors of early revision after anterior cruciate ligament reconstruction with hamstring autograft. Arthroscopy.

[21] Mariscalco MW, Flanigan DC, Mitchell J, Pedroza AD, Jones MH, Andrish JT, Parker RD, Kaeding CC, Magnussen RA (2013). The influence of hamstring autograft size on patient-reported outcomes and risk of revision after anterior cruciate ligament reconstruction: a Multicenter Orthopaedic Outcomes Network (MOON) Cohort Study. Arthroscopy.

[22] Middleton KK, Hamilton T, Irrgang JJ, Karlsson J, Harner CD, Fu FH (2014). Anatomic anterior cruciate ligament (ACL) reconstruction: a global perspective. Part 1. Knee Surg Sports Traumatol Arthrosc.

[23] Nagda SH, Altobelli GG, Bowdry KA, Brewster CE, Lombardo SJ (2010). Cost analysis of outpatient anterior cruciate ligament reconstruction: autograft versus allograft. Clin Orth Rel Res.

[24] Papastergiou SG, Konstantinidis GA, Natsis K, Papathanasiou E, Koukoulias N, Papadopoulos AG (2012). Adequacy of semitendinosus tendon alone for anterior cruciate ligament reconstruction graft and prediction of hamstring graft size by evaluating simple anthropometric parameters. Anat Res Int.

[25] Park SY, Oh H, Park S, Lee JH, Lee SH, Yoon KH (2013). Factors predicting hamstring tendon autograft diameters and resulting failure rates after anterior cruciate ligament reconstruction. Knee Surg Sports Traumatol Arthrosc.

[26] Pichler W, Tesch NP, Schwantzer G, Fronhofer G, Boldin C, Hausleitner L, Grechenig W (2008). Differences in length and cross-section of semitendinosus and gracilis tendons and their effect on anterior cruciate ligament reconstruction: a Cadaver study. J Bone Jt Surg.

[27] Pinheiro LF, de Andrade MA, Teixeira LE, Bicalho LA, Lemos WG, Azeredo SA, da Silva LA, Gonzaga LG (2011). Intra-operative four-stranded hamstring tendon graft diameter evaluation. Knee Surg Sports Traumatol Arthrosc.

[28] Prodromos C, Joyce B, Shi K (2007). A meta-analysis of stability of autografts compared to allografts after anterior cruciate ligament reconstruction. Knee Surg Sports Traumatol Arthrosc.

[29] Reboonlap N, Nakornchai C, Charakorn K (2012). Correlation between the length of gracilis and semitendinosus tendon and physical parameters in Thai males. J Med Assoc Thai.

[30] Samuelsson K, Andersson D, Ahlden M, Fu FH, Musahl V, Karlsson J (2013). Trends in surgeon preferences on anterior cruciate ligament reconstructive techniques. Clin Sports Med.

[31] Schwartzberg R, Burkhart B, Lariviere C (2008). Prediction of hamstring tendon autograft diameter and length for anterior cruciate ligament reconstruction. Am J Orthop.

[32] Schwartzberg RS (2014). Prediction of semitendinosus and gracilis tendon lengths and diameters for double bundle ACL reconstruction. Am J Orthop.

[33] Singhal MC, Gardiner JR, Johnson DL (2007). Failure of primary anterior cruciate ligament surgery using anterior tibialis allograft. Arthroscopy.

[34] Spencer EA, Appleby PN, Davey GK, Key TJ (2002). Validity of self-reported height and weight in 4808 EPIC-Oxford participants. Public Health Nutr.

[35] Thomas S, Bhattacharya R, Saltikov JB, Kramer DJ (2013). Influence of anthropometric features on graft diameter in ACL reconstruction. Arch Orthop Trauma Surg.

[36] Treme G, Diduch DR, Billante MJ, Miller MD, Hart JM (2008). Hamstring graft size prediction: a prospective clinical evaluation. Am J Sports Med.

[37] Tuman JM, Diduch DR, Rubino LJ, Baumfeld JA, Nguyen HS, Hart JM (2007). Predictors for hamstring graft diameter in anterior cruciate ligament reconstruction. Am J Sports Med.

[38] Xie G, Huangfu X, Zhao J (2012). Prediction of the graft size of 4-stranded semitendinosus tendon and 4-stranded gracilis tendon for anterior cruciate ligament reconstruction: a Chinese Han patient study. Am J Sports Med.

